# Talk to Me—Interplay between Mitochondria and Microbiota in Aging

**DOI:** 10.3390/ijms241310818

**Published:** 2023-06-28

**Authors:** Kristina Endres, Kristina Friedland

**Affiliations:** 1 Department of Psychiatry and Psychotherapy, University Medical Center of the Johannes Gutenberg-University, 55131 Mainz, Germany; 2 Department of Pharmacology and Toxicology, Institute for Pharmaceutical and Biomedical Sciences, Johannes Gutenberg-University, 55128 Mainz, Germany

**Keywords:** archaea, bacteria, fission, fusion, organelle, respiratory chain, virus

## Abstract

The existence of mitochondria in eukaryotic host cells as a remnant of former microbial organisms has been widely accepted, as has their fundamental role in several diseases and physiological aging. In recent years, it has become clear that the health, aging, and life span of multicellular hosts are also highly dependent on the still-residing microbiota, e.g., those within the intestinal system. Due to the common evolutionary origin of mitochondria and these microbial commensals, it is intriguing to investigate if there might be a crosstalk based on preserved common properties. In the light of rising knowledge on the gut–brain axis, such crosstalk might severely affect brain homeostasis in aging, as neuronal tissue has a high energy demand and low tolerance for according functional decline. In this review, we summarize what is known about the impact of both mitochondria and the microbiome on the host’s aging process and what is known about the aging of both entities. For a long time, bacteria were assumed to be immortal; however, recent evidence indicates their aging and similar observations have been made for mitochondria. Finally, we present pathways by which mitochondria are affected by microbiota and give information about therapeutic anti-aging approaches that are based on current knowledge.

## 1. Introduction

### 1.1. Common Evolutionary History of Mitochondria and Bacteria

Mitochondria are organelles with their own genome that arose from a proteobacteria living within single-celled archaea more than a billion years ago. The step of endosymbiosis offered tremendous opportunities for energy production and metabolism and allowed the evolution of fungi, plants, and animals.

Mitochondria share many similarities with prokaryotic cells, such as a double membrane, the ability to produce ATP through the generation of a proton gradient generated across the inner mitochondrial membrane, and the fact that they have their own genome and bacterial-type ribosomes. The mitochondrial DNA (mtDNA) found in mammals encodes 37 genes comprising 2 ribosomal, 22 transfer RNA, and 13 mRNA encoding for subunits of the mitochondrial respiratory chain [1,2]. The biggest part of the mitochondrial proteome, approximately 1500 proteins, are encoded by the nuclear genome necessary for oxidative phosphorylation, other metabolic pathways, and transcription and translation in mitochondria. Therefore, a coordinated crosstalk between the nucleus and mitochondria is essential to adapt mitochondrial function to the current energy need of cells [3]. Mitochondria consist of four sub-compartments. The outer mitochondrial membrane (OMM) is permeable to small molecules (<5 kDa). The intermembrane space (IMS) sequesters numerous proteins acting as damage-associated molecular patterns, such as cytochrome c, apoptosis-inducing factor (AIF), and several pro-caspases. Thirdly, the inner mitochondrial membrane (IMM) contains the different complexes of the respiratory electron transport chain (ETC, complexes I–IV) as well as the Fo-F1 ATP synthase (complex V). The ETC is responsible for the mitochondrial membrane potential (MMP). The IMM is rather impermeable due to cardiolipin, a phospholipid found exclusively in the inner mitochondrial and bacterial plasma membrane. It contains less fluid, requiring an orchestrated import mechanism for proteins that are encoded in the nucleus. The surface of this membrane forms cristae to increase the efficacy of the ETC and the ability to produce adenosine triphosphate (ATP) in the matrix. The matrix contains multiple copies of mtDNA organized into nucleoids as well as the machinery that is necessary to transcribe and translate mtDNA-encoded genes. Reducing agents (NADH and FADH2) are also generated in the matrix by the tricarboxylic acid (TCA) cycle and fatty acid ß-oxidation (FAO) [4].

Even if mitochondria still resemble in some parts their bacterial ancestor, the organelle also acquired new characteristics, such as a dynamic morphology of the mitochondrial network, which affects both mitochondrial activity and function. Mitochondria are very dynamic organelles moving along the cytoskeleton through cells to spots where energy is needed [5]. Furthermore, mitochondrial morphology is also permanently shaped by its surroundings, shifting from rounded/fragmented mitochondria into an interconnected and elongated tubular network. Mitochondrial function is maintained by a constant balance between mitochondrial fission and fusion [4,6,7,8,9,10,11]. Mitochondrial fusion is required to ensure mtDNA integrity. Depolarized and fragmented mitochondria are degraded by mitophagy, a specific form of autophagy. In healthy organisms, these two processes are balanced. Despite these adaptations as intracellular organelles, it is tempting to speculate on remnants of behavior of mitochondria derived from their evolutionary origin: can it be, that they still react to metabolites of the microbiota in a specific way, and does this contribute to physiology or vulnerability (as in aging) of the host? Competition and symbiosis can be considered when thinking of bacterial commensals, for example. Moreover, reactions to viruses similar to bacterial infection by bacteriophages is to be assumed. Interestingly, while eukaryotic viruses were found to be highly abundant in infancy, they decreased afterwards and remained constantly low for the rest of the life. Bacteriophages, on the contrary, decreased between adulthood and the elderly stage, with Siphoviridae matching best the overall trend [12].

### 1.2. Mitochondria in the Aging Organism

Mitochondria are semi-autonomous organelles acting as metabolic hubs in our cells. Mitochondria provide the majority of ATP via oxidative phosphorylation at the ETC. However, mitochondria are not only involved in energy metabolism; they also contribute to Ca^2+^ homeostasis, protein synthesis, metabolite synthesis, and programmed cell death via opening of the mitochondrial transition pore (mPTP), for example. Several recent reviews give an excellent overview regarding the role of mitochondria in aging, reflecting on every aspect mentioned above in detail [13,14,15,16,17,18,19]. Here, we briefly introduce the most important mitochondrial contributions to the aging process, which are also affected by bacterial metabolites or viral components.

During the aging process, mitochondrial function, especially mitochondrial membrane potential (MMP) and respiratory capacity per mitochondrion, decline [20,21,22]. This is often accompanied by increased production of reactive oxygen species (ROS) [23]. ROS are by-products of oxidative phosphorylation and are important signaling molecules. Mitochondrial dysfunction and reduced antioxidant detoxification result in oxidative stress, lipid oxidation, and mtDNA damage [24,25]. These observed changes in mitochondrial function and increased ROS production resulted in the free radical theory of aging [26]. However, recent findings suggest that balanced mitochondrial activity is essential for healthy aging. Recently, reduction in mitochondrial respiration and function was discussed as a protective mechanism leading to increases in lifespan. Several publications demonstrated that a mild reduction in MMP might result in the oxidized state of the ETC, leading to decreased ROS [27]. However, this mild depolarization was lost in different tissues of aging mice such as heart, muscle, and brain [17]. Therefore, constant loss of MMP as observed in the aging process might reflect reduced ETC capacity, reflected in the loss of activity and stability of ETC complexes during aging [23,28]. This decline is specific for different tissues with reduced complex III and IV activity in the heart muscle and diminished complex I and III function in brain, liver, and skeletal muscle, as well as parts of our immune system such as CD4+ T-helper cells [17,29,30]. Analysis of mitochondrial function in the skeletal muscle of older subjects revealed a drop in ATP levels [31,32] of approximately 50% compared to younger controls, which might also be the cause for sarcopenia typically associated with aging.

One reason for deficits in the mitochondrial respiratory chain is an altered mitochondrial stress response in aging and longevity. Since the mitochondrial proteome is encoded in both the nuclear and mitochondrial genome, mitochondrial protein homeostasis is central to mitochondrial function. Mild and time-restricted mitochondrial stress, such as disruption of most of the electron transport chain, results in the activation of the mitochondrial unfolded protein response (termed the mito-hormetic effect), which extends the life span in *C. elegans*, yeast, flies, and mice (for review please see [25]). In contrast, chronic OXPHOS dysfunction is often detrimental. Mitochondrial defects in mouse models result in a reduced lifespan, and human diseases associated with OXPHOS defects are typified by protracted defects [20,22,23,33].

Mitochondrial biogenesis is the process by which cells increase their individual mitochondrial mass in order to increase their energy production. Mitochondrial mass is also regulated by mitochondria-specific autophagy and maintenance of mitochondrial dynamics. Evidence of an age-related decrease in mitochondrial biogenesis has previously been reported, but the precise reason for this decrease remains elusive [34,35,36]. The PPARγ coactivator 1 (PGC-1) family, comprising of PGC-1α, PGC-1β, and PGC1-related coactivators, are main regulators of mitochondrial biogenesis. One important downstream target of PGC-1α are transcription factors such as TFAM (also involved in the regulation of mitochondrial biogenesis) or PPARγ [37,38]. Recent data indicate that impairments in mitochondrial biogenesis during aging may, at least in part, be a result of the age-related increase in mtDNA deletion, leading to a decreased ability to replicate mtDNA and to generate new mitochondria. Another factor discussed is mtDNA mutation [39,40,41,42,43]. It is well established that mitochondrial mutations increase during aging in both animal models and humans. These mutations differ in frequency between tissues and even within tissues in different cell types. The role of mtDNA mutations during the aging process is highly discussed because mtDNA exists in hundreds to thousands of copies in cells. It is generally accepted that the mutational load must exceed 60% of all mitochondria to lead to a phenotype [44]. Reduction in total mtDNA might be involved in mitochondrial dysfunction during aging [42].

Mitochondrial fission and fusion both contribute to dilution and segregation of damaged organelles, including mtDNA mutations, ensuring cellular homeostasis as well as survival after stress. Cellular and organismal health relies on a tight balance between mitochondrial fission and fusion processes [11]. A disruption in this balance is linked to aging, originally observed in lower organisms such as fungi or worms [20]. In mice, an impairment of both mitochondrial fission and fusion was observed during the aging process. In aged mice, reduced protein expression of the mitochondrial fission factor DRP1 seems to be a critical factor to reduce mitochondrial fission [45]. Again, the balance between fission and fusion seems to be the key to healthy aging because both knockdown and overexpression of DRP-1 results in muscle atrophy in 18-month-old mice [10]. In *C. elegans*, increased mitochondrial fusion is essential for longevity in the diverse longevity pathways, as inhibiting mitochondrial fusion reduces their lifespans to wild-type levels [46,47]. However, increased mitochondrial fusion is not a major driver of longevity but rather is essential to allow the survival of older animals beyond their normal lifespan in diverse longevity pathways [46].

When mitochondrial fission and fusion and other mitochondrial stress response pathways exceed their capacity to restore and repair mitochondrial function, mitophagy takes place [48,49,50]. This is the only mechanism which degrades mitochondria in total, thereby avoiding cellular damage and apoptosis. Mitophagy in higher eukaryotes operates in different tissues and cell types via a ubiquitin-dependent mechanism by the PINK-Parkin axis or a ubiquitin-independent mechanism via autophagy receptors such as BNIP3, NIX, and FUNDC1. Both pathways result in the engulfment of mitochondria in the autophagosome. Mitophagy reduction is observed in several tissues in mice. In a transgenic mouse model visualizing mitophagy with fluorescence-tagged mt-Keima, a reduction of 70% was discovered in the hippocampus of aged (21-month-old) mice compared to young (3-month-old) mice [51]. Furthermore, mitophagy is strongly impaired in the aging mouse heart, contributing to OXPHOS dysfunction and heart failure [52,53,54].

Aging alters cytosolic calcium handling [21,55] which may predispose mitochondria to calcium overload. This phenomenon is closely connected to the opening of the mPTP and initiation of apoptosis (for a comprehensive review please see [13]). The sensitivity of the mPTP to calcium is strongly enhanced by oxidative stress, reduced mitochondrial respiration culminating in ATP decline, and mitochondrial membrane depolarization. The regulation of mPTP opening is affected during aging as demonstrated in mitochondria isolated from different tissues such as brain, liver, or lymphocytes [56].

Pathological or accelerated aging in pre-frail and frail persons without other comorbidities is associated with so called “inflammaging”, characterized by higher circulating levels of inflammatory mediators such as CRP, IL-6, and fibrinogen [57,58,59,60]. One important finding is that the basal production of IL-6 in T-lymphocytes is higher; however, evoked responses to a cytokine challenge are reduced. This phenomenon is probably caused by a chronic activation of the JAK/STAT signaling pathway. A subset of elderly humans with higher rates of essential hypertension, arterial stiffness, and increased overall mortality is characterized by elevated IL-1ß levels and activation of the NLRP3 inflammasome [61]. Recent findings suggest that the chronic activation of NLRP3 inflammasome has deleterious effects which might be addressable by IL-1ß antibody caakinumab [62]. The immune system and mitochondria are connected via the release of mtDNA [58]. mtDNA acts as a damage-associated molecular pattern, activating NLRP3 inflammasome and driving caspase-1 activation and processing of pro-IL-1ß and pro-IL-18 [61,63]. NLRP3 is also regulated by mitochondrial ROS and the inner membrane lipid cardiolipin, a relic of the cell membrane of prokaryotic cells that gave rise to mitochondria [64].

### 1.3. Microbiota and Aging of the Host

The human gut-resident microbiota is composed of bacteria, archaea, protista, fungi, viruses, and phages [65]. Its composition is clearly affected by several diseases or nutritional and lifestyle conditions of the human host (e.g., increased *Ruminococcus gnavus* in irritable bowel syndrome, [66]). However, definition of the healthy human intestinal microbiome still is questionable [67]. This makes it rather difficult to evaluate which changes observed in aging, a naturally occurring phenomenon that involves highly variable functional decline [34,36,68], are deleterious and which are second to disabilities such as chronic inflammation or loss of barrier integrity. A meta-analysis including >2500 metagenomic datasets derived from individuals aged 20 to 89 years could segregate groups of bacterial taxa that were altered with disease per se and such that were altered especially in the elderly with disease [34]. Moreover, some bacterial metabolic pathways were found to correlate with frailty and not only with chronological age, such as choline consumption and trimethylamine production, which can be conducted by *C. clostridioforme*, for example. Thus, “usual suspects”, as they were designated in a recent review [69], might exist that foster unhealthy or healthy aging. Reduction in *Roseburia* and *Faecalibacterium* seems common in aging, while the increase in certain taxa seems to tip the scales: while *Akkermansia* and *Odoribacter* are mainly found associated with healthy aging conditions, *R. gnavus* or the genus Enterobacteriaceae have been associated with unhealthy aging (reviewed in [69]). For the effect of the host’s age on the gut fungal community, the mycobiome reports are scarce. In a study on Sardinians, the observed fungal communities could not be separated by age of the host [70]. Additionally, putative gut commensal fungal species with pathobiont potential were not found to differ between aged and young SPF mice [71]. Additionally, reports on archaea are not abundant: for example, an increase in the general amount was observed in centenarians in comparison to elderly and young subjects but did not reach statistical significance [72]. For protista, data are also limited. However, recently a correlation between Blastocystis, a single-cell eukaryotic microorganism frequently found that is capable of asymptomatic long-term host colonization, and cognitive performance in humans, was described [73]. Prevalence in a non-clinical population cohort was 30%, and interestingly, the age of subjects was associated with Blastocystis subtype carrier status which then influenced the bacterial community within the gut [74]. A meta-analysis considering 2697 metagenomes from 32 studies revealed a highly personalized virome and demonstrated that data also are severely dependent on the methodology used in the respective studies [12]. Nevertheless, the analysis concluded that a significant decrease in the richness of the virome occurs between adults and the elderly in healthy, western cohorts. This finding could be confirmed in a Chinese cohort. More recently, it was shown that richness of the gut virome increases again in centenarians compared to young but also aged (<60 years) adults when investigating samples from 195 Japanese and Sardinian individuals [75]. Namely, viruses associated with Clostridia were affected and higher lytic activity was found. Together with the microbial population, the centenarian virome seemed to support hydrogen sulfate output, which might also affect mucosal integrity, resistance to pathogens, and mitochondrial function [76]. Other reports draw an inconclusive picture on specific viral groups in the elderly: Alharshawi and colleagues [77] recently showed an increase in dUTPase antibodies, indicative of the Herpes virus, in plasma (comparing a group aged approximately 47 years and a group aged on average 66 years), while another report determined no correlation between age and RNA abundance in the Epstein–Barr virus (EBV, belonging to the Herpes virus family) with age [78].

To sum this up, the bacterial origin of mitochondria is widely accepted and both microbiota and mitochondria seem to be driving forces of healthy and diseased aging of their host. This poses the question of whether they comprise two independent factors or if there is—maybe due to their evolutionary entanglement—the possibility of a bacterial or microbial influence on the host mitochondria alongside aging. In the following chapters, we determine whether common processes of aging occur in bacteria and mitochondria, which pathways are known for microbiota effects on mitochondria, and attempt to deduce therapeutic options that may be used (via these interactions) to ameliorate deterioration by aging.

## 2. Aging of Bacteria and Mitochondria

**Figure 1:**
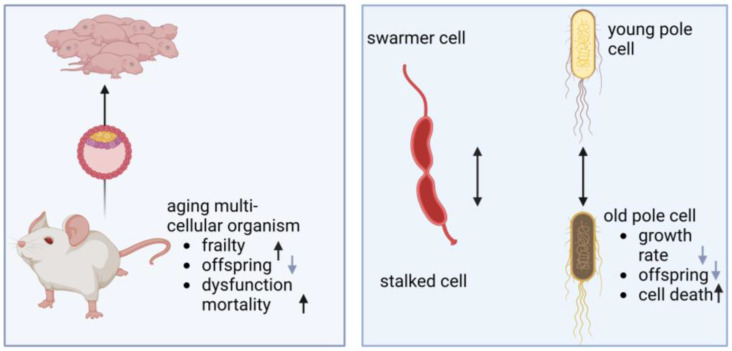
Aging in multi-cellular organisms and bacterial cells. Left: using germline cells, multi-cellular organisms such as mice allow their offspring to start with a non-exhausted cell pool while the parental animals accumulate dysfunctional cells with age, leading to diseased states, frailty, and finally death. Right: in bacterial cell division, morphological and functional asymmetry occurs. The schematic diagram of *C. crescentus* shows that during its life cycle, it gives rise to two morphologically different daughter cells: the stalked cell remains attached to the substrate, while the swarmer cell is motile due to its flagellum. *E. coli* and many other bacteria seemingly divide symmetrically. However, probabilities of the two cells rising from division, designated young and old pole cells, are divergent (image created by BioRender).

Bacteria were previously assumed to be immortal, escaping senescence via the consecutive formation of two viable and identical daughter cells with each division. However, in the last two decades the perspective has changed: several observations were made that indicate non-symmetrical divisions and with this, bacterial aging. Asymmetry was not only found in structural means, as with *Caulobacter crescentus* [79], but also in functional divergence of resulting cells. *E. coli* divides morphologically symmetrical; however, properties of the two cells derived from division are distinct and they can be distinguished as a “new pole” and an “old pole” cell (Figure 1). Stewart et al. demonstrated decreased growth rate and offspring production as well as increased incidence of death in the “old pole” cell, designating it as the parent cell that gave rise to juvenile offspring [80].

Similar to eukaryotic cells, stress seems to play a pivotal role in senescent bacteria. For example, the RpoS (RNA polymerase stress-response sigma factor) pathway, a general stress pathway, was found to correlate with the rate of aging in bacteria. Aged colonies even accumulate RpoS mutants that can evade the stationary phase and utilize acetate to continue growth [81], which makes it a sort of longevity factor in bacteria (reviewed in [82]). Contradictory findings regarding bacterial senescence have even been traced on the apparent stress factors (discussed in [83]).

The nine hallmarks of aging identified by Lopez-Otin in 2013 contain genomic instability, telomere attrition, epigenetic alterations, loss of proteostasis, deregulated nutrient sensing, mitochondrial dysfunction, cellular senescence, stem cell exhaustion, and altered intercellular communication [84]. These hallmarks have become a reference point for aging research in metazoans. For bacteria, unicellular organisms harboring circular DNA, at least stem cell exhaustion and telomeric shortening can be excluded. Intercellular communication can also be doubted as an important point in bacterial aging due to the unicellular occurrence. Nevertheless, bacteria can form biofilms bearing advantages for the community and rely on such communication. Biofilms present as very complex formations and reactions based on nutritional restriction cannot be excluded, which hampers analyzing aging-dependent changes in intercellular communication. Moreover, many investigations on aging in bacteria have been conducted in microfluidic devices, not allowing for communication between cells. Therefore, knowledge is rather limited in this regard (see [83]). Genomic instability as a contributor to aging could also not be demonstrated in bacteria without doubt (see Table 1). Probably, bacteria evolved too-efficient repair systems to counteract genomic instability including photoreactivation to reverse UV-induced damage or DNA alkylation, base excision repair and nucleotide excision repair, homologous recombination, non-homologous end joining, and single-strand annealing (reviewed in [85]).

For loss of proteostasis, on the contrary, examples in bacteria can be identified. Data on loss of proteostasis and aggregation of proteins seem more robust than those for other hallmarks of aging, but it has to be considered that bacterial inclusion bodies can also increase stress-resistance against external encounters [86]. An analogy can be drawn here regarding the aggregation of amyloid plaques in humans: approximately 40% in those aged 80 to 89 years were amyloid positive without having cognitive decline in a large population-based study [87]. Thus, increased protein aggregation can potentially be seen as an interim coping strategy with misbalanced production or degradation of unwanted proteinaceous products. Unfortunately, in humans the amyloid protein aggregation was correlated with increased incidence of developing mild cognitive impairment or even disease in the following years.

**Table 1:** Evidence for aging hallmarks in bacteria.

Hallmark	Investigation Tools	Organism	Observation	Ref
Genomic instability	fYFP-labeled MutL mismatch repair proteins	*E. coli*	Replication errors occur at a constant rate across ages.	[88]
	Growth arrest, DNA repair mutants	*E. coli*	Oxidative damage to DNA does not limit survival during stasis.	[89]
Loss of Proteostasis	Coomassie Blue-stained PAA gels	*E. coli*	Aggregates accumulate concomitantly with cell death. Dead cells contain increased amounts of aggregates. Aggregation occurs before cell death.	[90]
	Fluorescently tagged chaperone (IbpA)	*E. coli*	Aggregates migrate to the “old pole” cell.	[91,92,93]
	Fluorescently tagged chaperone (IbpA)	*E. coli*	Ejection of minicells with aggregates promotes stress resistancy of cells.	[94]
	Growth arrest	*E. coli*	Accelerated protein oxidation	[89]

Epigenetic modifications in bacteria consist of N6-methyl-adenine, C5-methyl-cytosine, or N4-methyl-cytosine, and the phosphorothioation of the DNA backbone might additionally lead to epigenetic marks (reviewed in [95]). Reports on aging and epigenetic regulation in bacteria are scarce. For *B. subtilis*, aging could be demonstrated; however, it did not correlate with sporulation, an epigenetic-regulated feature of these bacteria [96]. Further evidence for epigenetic modulation with bacterial aging is, to our knowledge, still lacking. Similarly, aging-dependent deterioration in nutrition sensing has not been described even if various sensor systems have been identified in bacteria (e.g., [97]). Interestingly, slowing down the growth rate of *E. coli* through the carbon source or via a carbon-limited chemostat reduced death rates during starvation afterwards [98]. This somehow resembles longevity-eliciting effects of caloric restriction in humans or animal models (for a recent review see [99]). We were not able to identify a publication reporting on the co-aging of the host and senescence of its commensal bacteria. It seems plausible that bacterial cells of lowered functionality might be expelled from the gut, for example. However, proteins involved in acetate conversion and utilization were upregulated during bacterial aging, and acetate can promote the persistence of mutated colonies that are capable of circumventing the stationary phase [100]. This was also observed for exogenous-administered acetate (not produced by the mutant strains themselves); thus, an altered microbiome might also prevent senescence of bacterial organisms by maintaining a comparably high acetate level.

Investigation of mitochondrial ultrastructure by electron microscopy unraveled two subpopulations of mitochondria gained from mitochondrial fission: those with a normal ultrastructure and mitochondria with a swollen morphology, indicating signs of damage [101,102,103]. Moreover, differences in transmembrane potential and opacity of mitochondrial content were observed as well as paracrystalline inclusion bodies formed of mitochondrial creatine kinase upon creatinine deficiency [104,105]. Interestingly, these subpopulations must not be equally distributed in the host cell. Under asymmetric division of stem-like cells from the human mammary gland, the “old” mitochondria are distributed unevenly across daughter cell populations [106], and this seemed mandatory for stemness. However, aging of mitochondria cannot be seen as clear as for bacteria, or even for eukaryotic cells, due to their specific biology: mitochondria not only undergo fission, but they also fuse, and this continuous and fast exchange of organelle content (“kiss and run”) can lead to preservation of a coherent population ([107], reviewed in [6]). A comparably low proportion of mitochondrial proteins (13 out of >1000) is encoded by the mitochondrion itself. Thus, the aging process of the host cell might easily override the aging of the organelle. Transient failures that might be seen as aging phenomena can, for example, be compensated by the unfolded protein response of the mitochondria (UPRmt). This process, however, depends on recognition of the damaged organelle by the host cell and on communication with the nucleus. This then downregulates protein import, for example, and with this, restores proteostasis of the organelle (reviewed in [108]). Another example is provided by the recently reported finding that TMBIM5 regulates dynamic reshaping of the mitochondrial proteome, guaranteeing proteostasis due to bioenergetic demands [109]. Potentially, the mere number of mitochondria per cell (ranging from ~80 to ~2000 in somatic cells, reviewed in [110]) and their potential to share and dilute defective components does not allow investigation into the aging of single organelles, and the mitochondria might better be seen as part of a multi-cellular organism despite their origin from unicellularity.

While cell fusion of eukaryotic cells occurs in the context of recombination or tissue repair regularly, it is restricted in regard to bacterial cells due to their cell wall. Nevertheless, they can shed the cell wall transiently due to environmental stress [111]. Prolonged exposure to the stressor leads to so-called L-forms. Fusion seems to benefit from membrane fluidity and interestingly, rigidity of membranes in L-forms increased with aging of the culture [112]. Thus, facilitated fusion in the mostly secure and homeostatic environment of the host cell might be an adaptation to circumvent aging processes by mitochondria. In highly differentiated host cells, fusion might be hampered by the expanded cytoarchitecture as it relies on motility of the organelles. Here, nanotunnels that have been found in mitochondria and bacteria might offer a way to maintain intercellular/interorganelle communication through the exchange of small molecules (review on mitochondrial nanotunnels: [113]). Whether this form of communication is affected by aging of either mitochondria or bacteria has, to the best of our knowledge, not yet been determined. An investigation on human skeletal muscle, however, related increased nanotunnels to mitochondrial stress and described a significant increase in patients with mtDNA-based diseases in comparison to healthy controls [114]. This might hint at the role of nanotunnels in the aging process.

## 3. Viral Impact on Mitochondria

**Figure 2:**
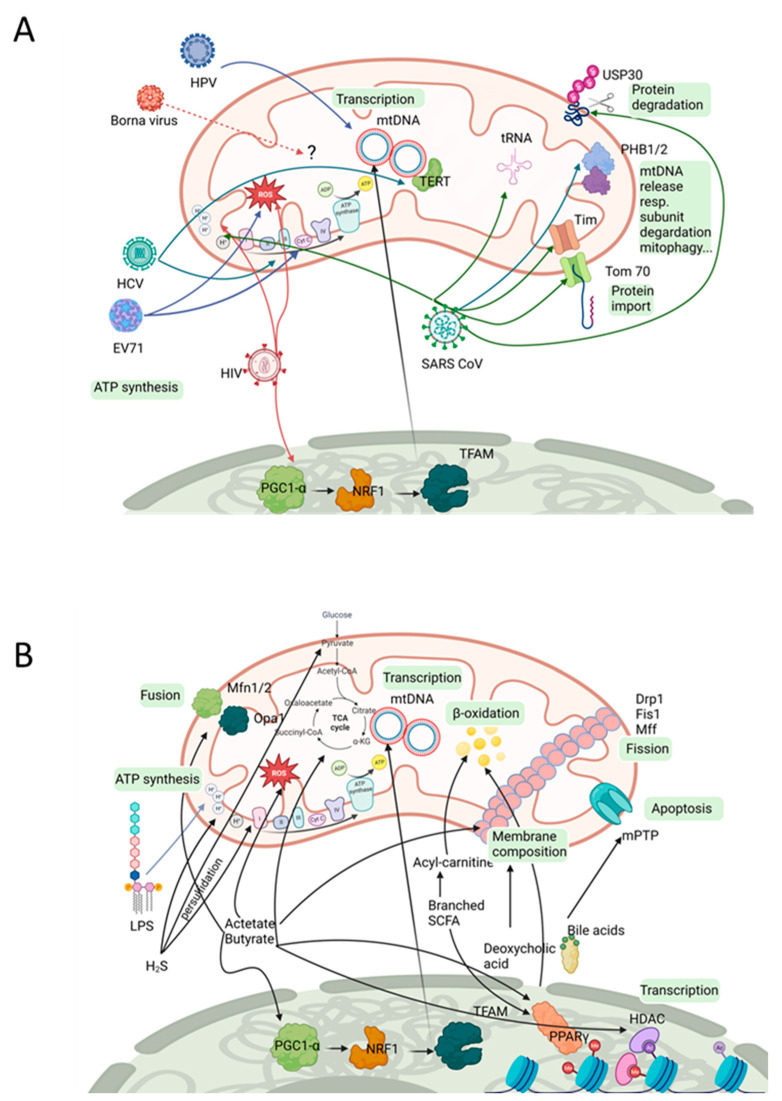
Affected mitochondrial pathways. Viral (**A**) as well as bacterial (**B**) commensals or pathogens can have an influence on a variety of molecular pathways that are important for maintenance and function of the host’s mitochondria. The impact of the microbiota might be directly or via the host’s own transcriptional/translational machinery, such as for PGC-1α (with subsequent activation of NRF1 and TFAM) or PPARγ (orchestrating lipid metabolism, e.g., β-oxidation) (the image was created by using BioRender). Drp1: dynamin-related protein 1; Fis1: mitochondrial fission protein 1; HDAC: histone deacetylas; Mff: mitochondrial fission factor; Mfn1/2: mitofusin 1/2; mPTP: mitochondrial permeability transit pore; NRF1: nuclear respiratory factor 1; Opa1: optic atrophy-1; TFAM: mitochondrial transcription factor A.

The gut microbiome contains a high density of organisms. While numbers might be difficult to compare, some organisms seem to have low abundance (0.1–0.8% of DNA content, reviewed in [65]) while bacteria dominate, making up approximately 93% of the DNA content. Viral DNA contributes 6% to the community. Organization and balance of the microbial community enforces communication. Specific ecological niches exist, built by the several longitudinal but also cross-sectional gradients within the gut (nutrients, oxygen, pH; [115]) that might allow specialists to occupy it without too much rivalry. However, when pathogens try to enter the already-studded space, several scenarios of viral/bacteria crosstalk can be considered. Firstly, co-infections have been observed where at least one of the organisms seem to profit from the precedence of another. One example is the infection by transmissible gastroenteritis virus (TGEV) that was shown to promote motility, invasiveness, and adhesion properties of *Enterococcus faecalis* and enterotoxigenic *E. coli* K88 (review on bacterial and viral intestinal co-infection; [116]). Secondly, competition for ecological niches can be considered. In 1965, the antibacterial activities of several viruses, such as *Herpes simplex*, were demonstrated [117]. Bacteria can themselves stabilize the virion, but they can also prevent viral infection as in the case of rotavirus infection of mice and segmented filamentous bacteria (interactions summarized in [118]. Here, the resource of the host cell is valuable, and it is good that hostile communication is deployed. This can be carried to extremes when bacteriophages come into play. A direct invasion of the bacterium is the goal and aggressive strategies are used. A phage–bacterial ratio of ∼1:1 has been suggested for the human gut, thus their impact on controlling and shaping the bacterial community should not be underestimated [65]. In sum, a wide spectrum of communication between viruses and bacteria has been established during evolution and leads to the assumption that remnants of these interactions might also be found in a mitochondria–virus interplay. Indeed, screening of the literature identified several viruses that interfere with mitochondrial function or other properties (see Table 2 and Figure 2). Some of the observed effects might rely on indirect effects elicited by influences of the host cell, such as in the case of the HIV-1 viral protein R (Vpr) that severely interferes with the axonal transport of mitochondria by modulating the host cell’s cytoskeleton [119]. Similarly, Hepatitis C virus (HCV) infection led to a mitochondrial localization of the host catalytic part of telomerase within the tumorous and peri-tumorous tissue of human patients (TERT, [120]), where it has no canonical function. The effect of this unusual localization has not been analyzed yet. However, there are also many cases in which the viral compounds directly localize within mitochondria and affect their function, and mitochondrial targets have been identified. For example, the nonstructured protein Orf9b from SARS-CoV binds to Tom70 [121]. This protein is one of the major import receptors in the translocase of the outer mitochondrial membrane. Co-immunoprecipitation indicated a direct interaction of Orf9b and Tom70, while other Tom complex members or other viral proteins failed. The Cryo-EM structure of the Tom70-ORF9b complex revealed that ORF9b binds to the Tom70 substrate binding pocket and with this might hamper the import of selective substrates such as PTEN induced kinase1 (PINK1) [122]. Regarding the localization of Tom70 in the outer mitochondrial membrane, it still must be accounted to the “host-side”. However, interaction of viral proteins with the mitochondrially encoded 4L subunit of NADH dehydrogenase or the TIM (translocase of the inner membrane) complex have also been described or at least have been predicted [121,123]. This opens up the possibility that here, old hostile or coexistence strategies between viruses and bacteria/mitochondria are the basis of these observations.

## 4. Eukaryotic Microbial Organisms with Mitochondrial Effectors

Alongside viruses and prokaryotic organisms, anaerobic/microaerophilic eukaryotes (protista and fungi) can be found in the intestinal system and are sometimes of high clinical relevance, such as *Entamoeba histolytica* or *Giardia intestinalis*. Under the oxygen-restricted conditions provided by the gut lumen, it seems implausible to maintain organelles with the main function of deriving energy from oxidative phopshorylation. However, the mitochondria of such organisms have been subjected to extensive reductionary processes and are termed mitochondrion-related organelles (MROs, reviewed in [138]). Entamoeba, MROs, for example, even lack the characteristic cristae. Depending on the ability to build ATP and on the substrates used as electron acceptors, they have been classified as aerobic mitochondria, anaerobic mitochondria, H_2_-producing mitochondria, hydrogenosomes, or mitosomes (classes 1–5, [139]). Nevertheless, these organisms share at least rudimentary pathways with the host cells via the ancestral characteristics of the mitochondria. This might limit their ability to interfere with the host’s mitochondria in a non-beneficial way. We were only able to identify two reports on *Trypanosoma cruzi* infection and its impact on the host’s cardiac mitochondria [30,140]. Carbonylation of subunits of mitochondrial respiratory complexes were observed in infected murine hearts as well as increased release of mitochondrial free radicals. Interestingly, mitochondrial morphology of some fungi seems to affect virulence in the case of pathological species such as *Cryptococcus deuterogattii* where a more tubular mitochondrial shape was correlated with an enhanced intracellular parasitism rate (reviewed in [141]).

## 5. Bacterial Compounds Affecting Mitochondria

One way for bacteria to communicate with host mitochondria is through bacterial metabolites [142,143,144,145]. Resistant starch is a type of feedstock which cannot be degraded by our enzymes. Other sources are cellulose, pectins, and oligosaccharides or amino acids such as valine, leucine, or isoleucine. Important fractions which are metabolized by microbiota in our gut are short chain fatty acids (SCFA) which contain up to seven carbon atoms, a straight-branched chain, and include formate, acetate, propionate, butyrate, isobutyrate, valerate, isovalerate, 2-methylbutuyrate, hexanoate, and heptanoate [145,146,147]. Fermentation of pectins and xylans lead to acetate, while starch is metabolized into butyrate [148]. SCFAs modulate oxidative status in the colon as well as mucosal inflammation, transepithelial fluid transport, and reinforce the epithelial defense barrier [149]. Importantly, the effects of SCFAs are not limited to the colon, but they may also affect mitochondrial function in the liver, the brown adipocytes, and skeletal muscles via G-Protein coupled receptors or FFA receptors [149,150,151,152]. The most abundant SCFAs are acetate, butyrate, and propionate, which are normally found in concentrations up to 150 mM in a ratio of 3:1.1 [153].

Acetate is produced by a wide range of Gram-negative microbes such as Bacteroides, Bififobacterium, Clostridium, or Ruminococcus species [148]. Acetate is converted to Acetyl-CoA by directly entering the TCA cycle (for a summary of mitochondrial pathways affected by viral and bacterial compounds see Figure 2). From all SCFAs, acetate reaches the highest concentration in the peripheral blood, contributing to energy production in the muscles, heart, and adipose tissue [146]. In the liver, acetate is used to produce energy, cholesterol, long-chain fatty-acids, glutamine, and glutamate [153,154].

Mainly Gram-positive anaerobic bacteria, such as Faecalibacterium or Eubacterium and Roseburia species from Clostridial clusters of Firmicutes, generate butyrate [146,147,155,156]. Despite being less abundant, butyrate is the main energy source for intestinal epithelial cells via feeding into mitochondrial respiration via the TCA cycle. In addition, it also interacts with G-Protein-coupled receptors GPR41, GPR43, and GPR109a, as well as with histone deacetylases (HDACs) [146,151]. Targets modulate a variety of intracellular signaling pathways and responses explaining the pleiotropic anti-inflammatory effects of butyrate. The inhibition of HDACs by butyrate also stimulates mitochondrial biogenesis and thereby energy expenditure. In addition, butyrate promotes physiological hypoxia in colonocytes by stabilizing the hypoxia-inducible factor (HIF) [157,158]. HDACs and HIF both serve as barrier-protective genes and influence mucosal homeostasis. Butyrate reduces gut mucosal inflammation by increasing the production of anti-inflammatory cytokines such as transforming growth factor β (TGF-ß), IL-10 secretion from T-cells, and IL-18 secretion in the intestinal epithelium [159]. In addition, butyrate inhibits the production of pro-inflammatory signaling molecules such as the nuclear factor kappa B (NF-ΚB) [160].

Both metabolites prevent the down-regulation of mitochondrial fusion factors and up-regulation of fission factors in pancreatic beta cells after treatment with the apoptosis inducer and metabolic stressor streptozotocin at rather high concentrations. Acetate showed more efficiency in enhancing metabolism and inhibiting ROS, while butyrate had less effect but was stronger in inhibiting the SCFA receptor GPR41 and NO generation [149]. Another protective mechanism in colonoctyes, discussed for both metabolites, is the activation of AMP-activated protein kinase (AMPK). AMPK is an essential energy sensor for mitochondrial oxidative phosphorylation and regulates gene expression resulting in reduced lipogenesis and an increase in the AMP:ATP ratio [161,162,163].

Propionate is also produced by Gram-negative phyla Bacteroidetes and Actinobacteria in the intestinal tract and is considered to promote mitochondrial activity and metabolic reprogramming [147,164].

Branched SCFAs, such as isobutyrate, 2-methylbutyrate, and isovalerate, are produced during the breakdown of proteins under a decreased fiber supply [146]. They elevate acylcarnitine levels, indicating an aberrant fatty acid oxidation, and promote mitochondrial biogenesis by activating PGC1α and its downstream target PPARγ [165,166].

Alongside SCFA, bacteria produce secondary bile acids [164,167]. Anaerobic bacteria, e.g., Eubacterium, Clostridium, and Bacteroides, degrade 5–10% of primary bile acids into secondary bile acids. They engage with the nuclear receptor, the transcriptional regulator farnesoid X receptor (FXR), and the GPCR TGR5 [168], and activate downstream targets such as PPARγ or other transcriptional regulators such as the epigenetic regulator SIRT1 or the hormone fasting-induced adipose factor FIAF. They thereby modulate carbohydrate (SIRT1, FIAF) and lipid metabolism, increasing fatty acid oxidation and oxidative phosphorylation. They also directly modulate mitochondrial function and biogenesis. Deoxycholic acid impairs membrane fluidity and is discussed to alter mitochondrial membrane composition [169]. Hydrophobic secondary bile acids induce apoptosis via formation of the mitochondrial transition pore (mPTP) [170].

Mitochondria also metabolize proteins and amino acids. Here, two amino acids are of special interest: the non-essential amino acid cysteine and the essential amino acid tryptophan. Microbiota, e.g., *Escherichia coli*, are able to produce tryptophan which then can be made available to the host [171]. Tryptophan is the precursor to the neurotransmitter serotonin and plays a role in synthesizing the cofactors for redox reactions nicotinamide adenine dinucleotide (NAD) and NAD phosphate (NADP), both fuels for the electron transport chain of mitochondria. Other bacteria, e.g., *Streptomyces antibioticus*, *Cynaidium caldarium*, *Karlingia rosea*, and *Xanthomonas pruni*, use tryptophan to produce quinolinic acid, which is a neuroactive metabolite of the kynurenine pathway. Cysteine is a component of the antioxidant glutathione detoxifying mitochondrial ROS, and is metabolized into H_2_S by intestinal cells and microbiota such as *D. desulfuricans*, Desulfobacter, Desulfobulbus, and Desulfotomaculum [172]. H_2_S shows bell-shaped effects on cellular functions in lower concentrations supporting cellular bioenergetics, whereas higher concentrations block cellular respiration [146,173]. In low concentrations, H_2_S primes the mucosal barrier functions and protective immunity. H_2_S stimulates mitochondrial bioenergetics by feeding into the mitochondrial electron transfer chain and mediating persulfidation of ATPase and glycolytic enzymes. In contrast, excessive H_2_S production by Fusobacterium is associated with intestinal inflammation. It induces DNA-damage in epithelial cells, inhibits SCFA metabolism, and compromises the mucus barrier by inducing breaks and permitting exposure of luminal contents to the underlying tissues. Another gas produced by mitochondria is NO. NO interacts at physiological levels with the reduced heme center of the cytochrome c oxidase, thus competing with oxygen binding or at the oxidized copper center by nitrite. In higher concentrations above 1 µM, NO, as well as peroxynitrite, inhibit mitochondrial function unselectively but irreversibly due to modification of proteins. However, NO also stimulates mitochondrial biogenesis via the PGC-1α pathway.

Another important bacterial component affecting mitochondria are lipopolysaccharides (LPS), a major component of the Gram-negative bacterial outer membrane [174]. LPS binds to toll-like receptors (TLR), mainly TLR4, which activate NF-KB and upregulate inflammatory chemokines and cytokines [175,176]. LPS injections into animals resulted in reduced complex I, II, and IV activity and mitochondrial uncoupling [177].

Trimethylamine-N-oxide (TMAO) is produced through meta-organismal steps from precursors such as betaine and its metabolites γ-butyrobetaine and choline, which are converted to trimethylamine (TMA) by the gut microbiota belonging to Firmicutes and Proteobacteria phyla in the intestine. TMA is absorbed through the intestinal wall and transported to the liver and oxidized into TMAO. TMAO increases the expression of pro-inflammatory IL-6 gene and chemokine ligands such as TNFα [178].

During the aging process the profile of microbiota in the gut changes, as already pointed out in chapter 1.3, and the composition of metabolites is altered [67,69,179]. The metabolites butyrate and acetate are associated with younger age groups and healthy aging groups. Metabolites such as TMA, LPS, or deoxycholic acid are produced by unhealthy aging-associated pathobionts.

Most interactions between microbiota, such as bacteria, their metabolites, and the host body, occur where these microbial organisms are highly concentrated, e.g., the gastrointestinal tract. However, several bacterial metabolites, e.g., SCFAs, are discussed to not only act on the gut as the place of origin but also on the brain. In addition, bacteria also synthesize hormones, including glucagon-like peptides and neurotransmitters, such as GABA, serotonin, dopamine, or acetylcholine, which shape the function of the enteric nervous system and might also communicate via the vagus nerve with our brain. Several recent reviews give an excellent overview on the gut–brain axis and how this axis is altered in aging and neurodegenerative disease [180,181,182,183,184,185].

## 6. Deducing Therapeutic Interventions from Current Knowledge

To shift the microbiome which drives unhealthy aging to a microbiome which promotes healthy aging, several strategies, such as prebiotics, synbiotics, postbiotics, health-linked dietary regimes, and exercise, are discussed [69,186,187,188]. Prebiotics include nutritional supplements increasing beneficial microorganisms such as freeze-dried blueberry powder which consists of high levels of polyphenols. Consumption of blueberries or blueberry powder for six weeks increased butyrate-producing microbiota in aged women such as Faecalibacterium and Coprococcus as well as Butyricimonas and Barnesiella [189,190,191,192,193]. This microbiota shift was accompanied by an increase in antioxidant activity in baseline and placebo controls. Synbiotics are single or many beneficial microorganisms administered in combination with prebiotics. Postbiotics are deliberately inactivated microbial cells, cell components, or microbiota-derived metabolites. The aim of interventions with prebiotics and postbiotics is to increase beneficial taxa, such as Lactobacilli and Bifidobacteria, in addition to butyrate-producing bacteria and Akkermansia [69]. These taxa promote insulin sensitivity, maintain intestinal barrier activity, and cross-feed the butyrate producers with acetate. For example, administration of live or pasteurized *Akkermansia muciniphila* for three months in humans reduced body weight, insulin resistance, and cardiometabolic risk factors [194,195,196]. In addition, pathobionts such as Desulfovibrio, Streptococcus, and Enterococcus must be reduced to promote healthy aging.

Another and probably more effective concept to shift the microbiome toward healthy aging is whole-diet intervention [69]. One recent study focusing on this concept is the Mediterranean diet (MedDiet) intervention, increasing the intake of vegetables, fruits, legumes, fish, olive oil, and nuts and reducing the intake of red meat, dairy products, and saturated fats [197,198]. The effects of the MedDiet on the prevention of diseases such as cancer, cardiovascular diseases, or Alzheimer’s disease have been known for decades. However, until now, no data were available on how the MedDiet alters the microbiome. The study by a European consortium, NU-Age MedDiet, shows in 323 older intervention participants compared to 289 age matched controls that butyrate producers, e.g., *Faecalibacterium prausnitzii* or *Roseburia hominis*, were increased and pathobionts, such as *Clostridium ramosum*, were decreased. The pathobionts were also linked to greater genomic coding capacity for the production of harmful metabolites such as deoxycholic acid.

**Figure 3:**
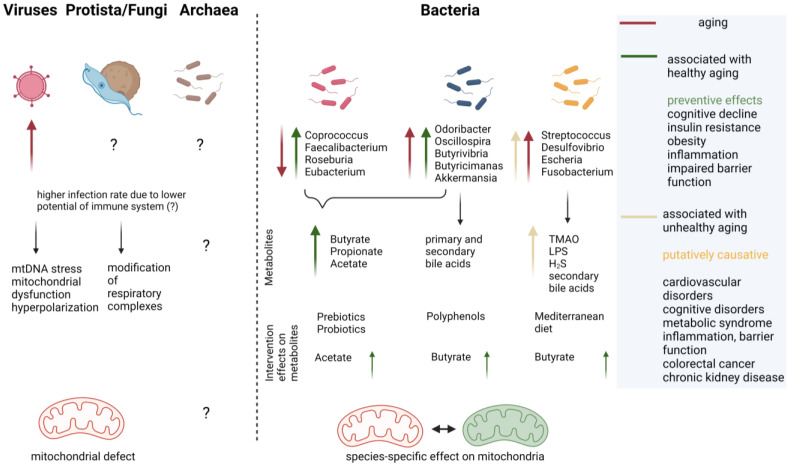
Changes in microbiota and their metabolites during aging and their protective role or their involvement in the pathophysiology of diseases. Effects of interventions such as prebiotics, probiotics, polyphenols, and Mediterranean diet on metabolites of microbiota are indicated (arrows indicate direction of change (increase or decrease); ?: unknown; the image was created by using BioRender).

Exercise to promote healthy aging is a hot topic and affects mitochondrial function by improving mitochondrial biogenesis and balancing mitochondrial fission, fusion, and mitophagy [176,177,178]. Furthermore, moderate exercise alters microbiota composition and increases *Akkermansia muciniphila* as well as Lactobacilli and Bifidobacteria, promoting epithelial barrier integrity and producing SCFAs. Aerobic exercise increases SCFAs and thereby alters luminal pH in the colon by decreasing primary bile acids to secondary bile acids such as deoxycholic acid. A summary of the above-reported influence of microorganisms is given in Figure 3.

Anti-viral drugs might exert another therapeutic pathway. Interferon administration in HC patients, for instance, lowered the frequency of mtDNA mutations in hepatocytes [199]. Direct eradication of HCV in a study including peripheral monocytic blood cells from 133 patients with a mean age of 66 revealed a restoration of mitochondrial function [200]. Maximal and reserve respiratory capacity increased as well as mtDNA integrity. To assess the risk of interferon treatment in the geriatric population, a population with high HC infection rates, a retrospective study was conducted including approximately 500 patients [199]. It revealed good therapeutic outcomes as well as similar survival rates and side effects when comparing early and late elderly individuals. Thus, targeting Herpes viruses seemingly presents a therapeutic option to target mitochondrial dysfunction in aging. An example where this seems unfeasible with the currently used drugs is antiretroviral therapy (ART) or highly active ART in HIV therapy. The antiviral thymidine analogues themselves lead to mitochondrial dysfunction and oxidative stress [200]. These nucleoside analogues might even be incorporated into mtDNA, leading to mutations or termination of mtDNA synthesis in combination with deregulation of DNA repair mechanisms, as reported in T cells, for example [200]. Very recently, activation of endogenous retroviruses in aged mice and primates, but also in human tissue and serum, was assessed and was suggested as a biomarker for aging [100]. Suppression of these viruses revealed anti-senescent effects.

Another, more fundamental option of interfering with the whole-gut microbiota is provided by the overwriting of the original community by installing an administered microbiota presenting better health conditions—or, in the case of aging, younger age. This fecal material transfer (FMT) is well established and is demonstrated to be highly efficient, for example, in the case of *Clostridium difficile* infections [201]. However, regarding aging, only pre-clinical data exist. For instance, it could be demonstrated that transfer of an aged wild-type microbiota into 5xFAD Alzheimer model mice aggravated certain aspects of pathology [201]. Moreover, transfer of an aged microbiota into wild-type mice resulted in altered expression of proteins related to synaptic plasticity or neurotransmission in the hippocampus and an aging-like phenotype of microglia within the hippocampus [202]. Efficacy of FMT as a therapeutic option for ameliorating age-dependent decline was also conducted in mice with transfer of young animal-derived material in aged individuals: the myeloid skew that is characteristic of aging could be mitigated and the hematopoietic stem cells could be rejuvenated [199]. In another study, restoration of *A. muciniphila* abundance (and with this, metabolite acetic acid) via fecal material from young mice displayed hepatoprotective effects and improved the antioxidative capacity, for example [199]. However, while these studies are promising, unfortunately, a detailed analysis of mitochondrial properties was not undertaken.

## 7. Conclusions

Aging is a phenomenon that seemingly affects all organisms—even microbial, single-celled ones. Thus, that organelles derived within evolution from these microbes are also subjected to the process is highly plausible. Nevertheless, in addition to sharing hallmarks of aging, due to their ancestral characteristics, mitochondria are also targets for microbes that might have shared an ecological niche with them in earlier times. For viruses and bacteria, direct interference or effects of metabolites in a more indirect manner can be identified that support or harm mitochondrial function. Knowledge on such interferences by protista, fungi, and archaea as part of the human microbiota is rather restricted and needs further investigation. These microbial–mitochondrial interactions can modify the aging process of the host where sustained energy provision might be needed to counterbalance decline of structures and functional complexes. This might be of special relevance for neuronal cells, as their long axons and high demands of energy do not allow even a subtle decline in energy support without leading to deterioration of function and development of neurodegeneration. Extended knowledge on the respective pathways by which our diverse commensals drive mitochondrial properties might finally provide future therapeutic approaches in fostering healthy aging by maintaining energy support.

## Figures and Tables

**Table 2:** Viral components affecting mitochondrial function.

Virus	Viral Component	Mitochondrial Component	Observation/Theoretical Impact	Ref.
Herpes virus	Whole virus	mtDNA	Virus infection induces mtDNA stress (increase in larger nucleoids, decrease in total nucleoid number).	[124]
SARS-CoV	5′/3′ untranslated regions contain putative mitochondrial residency sites		Localization in host mitochondria.	[125]
	Viral nonstructured protein Orf9b	Tom70	Localization in host mitochondria.	[121]
	Nsp5 (C145A)	tRNA methyltransferase 1 (TRMT1)	Enzyme for dimethylation of guanosine (m2,2G) on mitochondrial tRNAs.	[121]
	ORF9b		Localization in host mitochondria.	[126]
	ORF8a		Localization in host mitochondria, increases in mitochondrial transmembrane potential.	[127]
	Nonstructural protein 2 (Nsp2)	Prohibitin 1 (PHB1) and PHB2 (also other potential host interaction partners identified for Nsp1 and 2)	Mitochondrial biogenesis.	[128]
	ORF3a	Mitochondrial ubiquitin-specific peptidase 30 (USP30)		[129]
	Nsp10	NADH 4L subunit and cytochrome oxidase II	NADH cytochrome activity altered, inner mitochondrial membrane depolarized.	[123]
	Global analysis of SARS-CoV-2 host-interacting proteins: 26 of the 29 SARS-CoV-2 proteins) Nsp4, Nsp8, Orf9c, structural protein M	332 high-confidence SARS-CoV-2-human protein–protein interactions; with mitochondrial relevance: Tim complex, mitochondrial ribosome, electron transport	Mitochondrial function.	[121]
HIV-1	Tat (transactivator of transcription protein)	?	Hyperpolarization of mitochondria in cortical neurons.	[130]
	HIV-1 viral protein R (Vpr)	? (probably microtubule structure of the host cell)	Disturbed axonal transport. Reduced peroxisome proliferator-activated receptor-gamma coactivator 1 alpha (PGC-1α) expression via increase in the methylation of the PGC-1α promoter.	[119]
	Tat and negative-regulating factor (Nef)	?	Increased ROS production and mitochondrial mass destabilization of the mitochondrial membrane potential.	[131]
	Tat	Mitochondrial KATP channels discussed	Mitochondrial hyperpolarization.	[130]
	Infection	?	Increased mitochondrial ROS levels. Decreased ATP-linked respiration.	[132]
	HIV gp120 and Tat		Inhibition of mitophagic flux. Increased mitochondrial fragmentation. Increased sequestosome 1 translocation to damaged mitochondria.	[133,134]
Enterovirus 71 (EV71)	Infection	?	Decrease in mitochondrial electrochemical potential. ΔΨ(m) increase in oligomycin-insensitive oxygen consumption. Induced ROS production.	[135]
Borna disease virus (BDV)	Accessory protein X	?	Colocalizes with mitochondria.	[136]
Hepatitis C virus (HCV)	D2 domain of NS5A (NS5A-D2)	(might be due to glucokinase activation of the host cell)	Reduced spare respiration capacity.	[137]
	Infection	?	TERT localization in mitochondria.	[120]

?: targeted mitochondrial component unknown.

## Data Availability

No new data were created or analyzed in this study. Data sharing is not applicable to this article.
